# Optically reconfigurable polarized emission in Germanium

**DOI:** 10.1038/s41598-018-29409-3

**Published:** 2018-07-24

**Authors:** Sebastiano De Cesari, Roberto Bergamaschini, Elisa Vitiello, Anna Giorgioni, Fabio Pezzoli

**Affiliations:** LNESS and Dipartimento di Scienza dei Materiali, Università di Milano-Bicocca, via Cozzi 55, I-20125 Milano, Italy

## Abstract

Light polarization can conveniently encode information. Yet, the ability to tailor polarized optical fields is notably demanding but crucial to develop practical methods for data encryption and to gather fundamental insights into light-matter interactions. Here we demonstrate the dynamic manipulation of the chirality of light at telecom wavelengths. This unique possibility is enrooted in the multivalley nature of the conduction band of a conventional semiconductor, namely Ge. In particular, we demonstrate that optical pumping suffices to govern the kinetics of spin-polarized carriers and eventually the chirality of the radiative recombination. We found that the polarized component of the emission can be remarkably swept through orthogonal eigenstates without magnetic field control or phase shifter coupling. Our results provide insights into spin-dependent phenomena and offer guiding information for the future selection and design of spin-enhanced photonic functionalities of group IV semiconductors.

## Introduction

The ever-increasing demand for fast, secure data processing and transmission has sparked an intense search for high-volume and cost-effective approaches to integrate sensing^1^, storage^2,3^ and communication^4,5^ functionalities within semiconductor devices. Spin and photon degrees of freedom have emerged as competing candidates in the quest to extend or even substitute current electronics based on charge transport^6–8^. Their coupling, albeit challenging, holds the prospects for a new family of multifunctional, high-performance and energy-efficient devices^9–12^. Merging magnetic and photonic functionalities would open the way to a wide array of advanced operations encompassing (i) large-bandwidth and fast signal switching by polarization multiplexing in data transmission protocols^13–15^, (ii) optical writing of nanomagnets for high-density and high-speed memories^16–18^, (iii) encoding information for chip-scale and long haul optical fibre communication^19–21^, and (iv) lab-on-chip biological sensing of chiral-dependent structures and chemical phenomena^22–24^.

The absorption of circularly polarized photons and the resulting distribution of the spin angular momentum between the photo-generated carriers via the optical selection rules was exploited in the pioneering demonstration of ultrafast optical switching through coherent spin rotations^25^. This anticipated lasing dynamics exceeding 100 GHz^25–27^ and disclosed the potential of spin-optoelectronics to outperform its conventional counterpart^25–29^. Switching the state of light polarization, however, turns out to be a major hurdle. The lack of simple turning knobs is yet a crucial roadblock as current approaches mostly rely on strong external magnetic fields or on bulky phase shifters^11,25^.

This stimulated us to identify radically new concepts to achieve the on-demand definition of the angular momentum of the polarized component of the emission. In this exploratory work we address this challenge by demonstrating that Coulomb-mediated interactions can be optically tailored to precisely control the light polarization through the manipulation of the spin at selected minima or valleys of a semiconductor. Here we validate this concept by applying Stokes polarimetry to the low temperature direct gap photoluminescence (PL) of germanium^30^, whose multi-valleyed structure of the conduction band (CB) naturally suites best this purpose^31^. Notably, the optical properties of Ge match well a desired operation at telecommunication wavelengths^32^ and the recent demonstrations of optical gain^33–38^ make Ge a promising candidate also for the monolithic integration of light sources onto the conventional Si-based microelectronics platform.

In our experiments, the polarization of the direct gap PL was found to be continuously tunable between left- (|*L*〉) and right-handed (|*R*〉) circular polarization eigenstates. This was achieved by optical pumping at a fixed excitation energy and by solely modulating the laser excitation fluency. Moreover, we show that linearly polarized PL, i.e. 1/2 (|*L*〉 + |*R*〉), can be obtained as a consequence of a coherent superposition of two equal populations of electrons having opposite spin orientations.

## Results

### Concept of dynamical spin control

Before applying chiroptical spectroscopy, we illustrate the intriguing spin dynamics that pertains to the multivalley CB of Ge and elaborate on the proposal of its possible exploitation to attain an unmatched polarization control.

**Figure 1:**
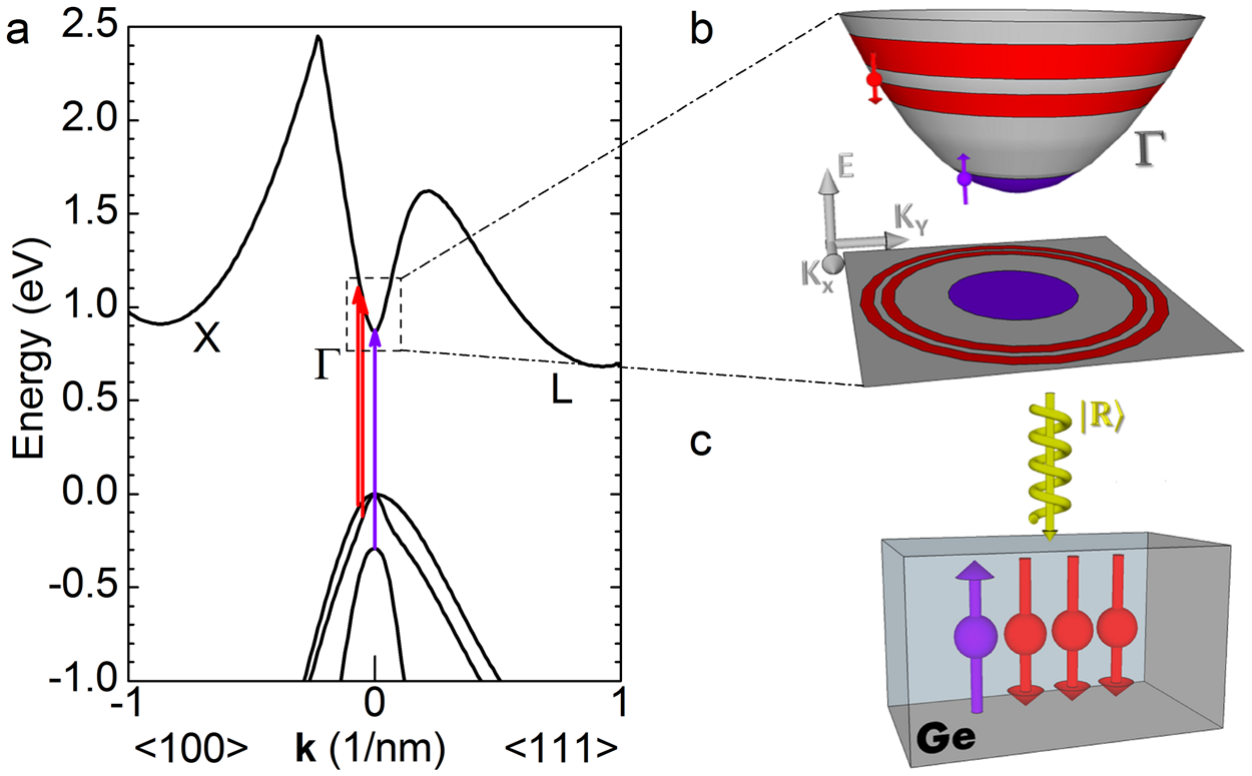
Optical orientation process in Ge. (**a**) Band structure of Ge along the [111] and [100] directions. (**b**) Schematic of the spin and momentum distribution of conduction band electrons located at the Γ valley. (**c**) Sketch of the geometry of the optical orientation experiment in Ge.

In Ge, the excitation via infrared circularly polarized light with energy almost resonant with the direct gap threshold results in optical spin orientation (see Fig. 1a,b)^30,39^. Vertical transitions in the vicinity of the Γ point at the centre of the momentum space create a non-equilibrium population of spin-polarized electron and hole pairs characterized by the quantum numbers |*J*, *J*_*z*_〉 describing the total angular momentum, *J*, and its projection, *J*_*z*_, along the quantization axis. Under our experimental conditions, light impinges perpendicularly onto the (001) sample surface and the spins point towards the *z* direction defined by the photon momentum (see Fig. 1c).

By virtue of spin orbit coupling and of the dipole-allowed selection rules, two subsets of electrons can be notably initialized in the CB with different kinetic energies and with spins pointing in opposite directions (Fig. 1b). In particular, upon |*R*〉 excitation the largest fraction of electrons has a sizeable momentum and, on average, a spin-down direction, i.e. |1/2, −1/2〉, being optically coupled chiefly to the upper heavy hole (HH) valence band (VB) states, |3/2, −3/2〉^6,40^. Simultaneously, a few percent of the whole electron ensemble can be promoted at the edge of the Γ valley as |1/2, 1/2〉 spin-up electrons via transitions involving split-off |1/2, −1/2〉 VB states (Fig. 1a,b)^6,40,41^.

In the process of reaching thermal equilibrium with the crystal, the photogenerated holes relax towards the top of the VB at Γ, while electrons sample the minima landscape of the CB. The latter results in puzzling spin-dependent phenomena and represents a remarkable difference with respect to the well-known case of direct gap materials like III-V compounds, where |1/2, ±1/2〉 electrons can simply thermalize at Γ^30,31^.

In indirect gap materials, electrons ultimately scatter out of the optically coupled Γ region and are eventually collected at the global minima. In Ge these are energy-degenerate and located along the four equivalent [111] directions at the *L* point of the Brillouin zone as shown in Fig. 1a.

It is worth noting that in Ge the additional satellite *X* valleys, owning to a large density of states, offer a preferential kinetic pathway for the spin-down electrons generated above the edge of the Γ valley. Literature works pointed out the importance of the energy relaxation channel opened by the *X* valleys^42,43^ and the Coulomb interactions occurring therein^30^. The change in the effective mass of the photogenerated electrons transferred from the Γ to the *X* valleys manifests itself in an enhanced scattering probability with plasmon and background carriers. Such cooling mechanisms are more effective than phonon-mediated processes, e.g., Frohlich or deformation potential interactions^30^. The latter are indeed efficient in polar III-V semiconductors but not in Ge, owning to its covalent centrosymmetric crystal structure^30^. In highly-doped Ge samples, collective and binary collision processes with the Fermi-Dirac distributed background carriers have been argued to particularly enhance the occurrence of scattering events guiding a minute fraction of spin-down electrons back to the original Γ valley^30,44^. This mechanism, in turn, is meant to cause a reversal of the direct gap circular polarization with respect to the case of weakly-doped samples, whose band-edge luminescence is determined by the pristine lower-energy spin-up electrons^30^. It should be noted that spin-orbit coupling mixes the spin-dependent wavefunctions of VB Bloch states, typically resulting in an unpolarized hole ensemble^45,46^. On the contrary, the spin relaxation time of electrons greatly exceeds the lifetime at Γ and thus dictates the state of the direct gap PL polarization^31,47^.

While above 30 K the spin relaxation of *L*-valley electrons is expected to be driven by zone-edge intervalley electron phonon coupling^48^, literature data disclosed the opening of extrinsic spin relaxation channels, possibly related to impurities, that already below 60 K outweigh the intrinsic Elliott-Yafet mechanism^31,47^. While this shortening of the spin lifetime can be mitigated in low dimensional heterostructures^47^, in bulk Ge the spin relaxation of conduction band electrons remains below 200 ns as the lattice temperature approaches 4 K. Following this line of reasoning, the polarization of the direct gap PL is likely to be unaffected by the abovementioned impurity and intervalley scattering since the dynamics at Γ occurs on a much faster ps time scale than spin relaxation at the *L*-valley^42^. Although a simple consideration regarding the density of states implies that the *L*-valley population is several orders of magnitude larger than the one at the zone center, *L*-valley carriers are also unlikely to have sizeable effects on the kinetics of the Γ-valley spin polarized electrons because of the large difference in momentum and because at low temperatures *L*-valley electrons cannot experience backward scattering towards the zone center^30^.

Inspired by these findings, we can explore the unique possibility to utilize the effective momentum-spin locking of the photo-injected electrons to actively manipulate the dwelling of a selected spin ensemble at the zone centre, thereby altering the spin imbalance accumulated at the direct valley through the initial optical orientation process. In the following, we leverage photo-injection to intentionally modify the free-carrier concentration. By doing so, we demonstrate how to optically alter the occurrence of Coulomb-mediated interactions and achieve the dynamical control over the PL polarization.

### Stokes polarimetry applied to Ge

We focus now on Ge:As wafers whose doping concentration is 8.3 × 10^16^ cm^−3^. As it will be discussed in the following, the choice of the doping level and lattice temperature facilitates the successful control of the polarization.

**Figure 2:**
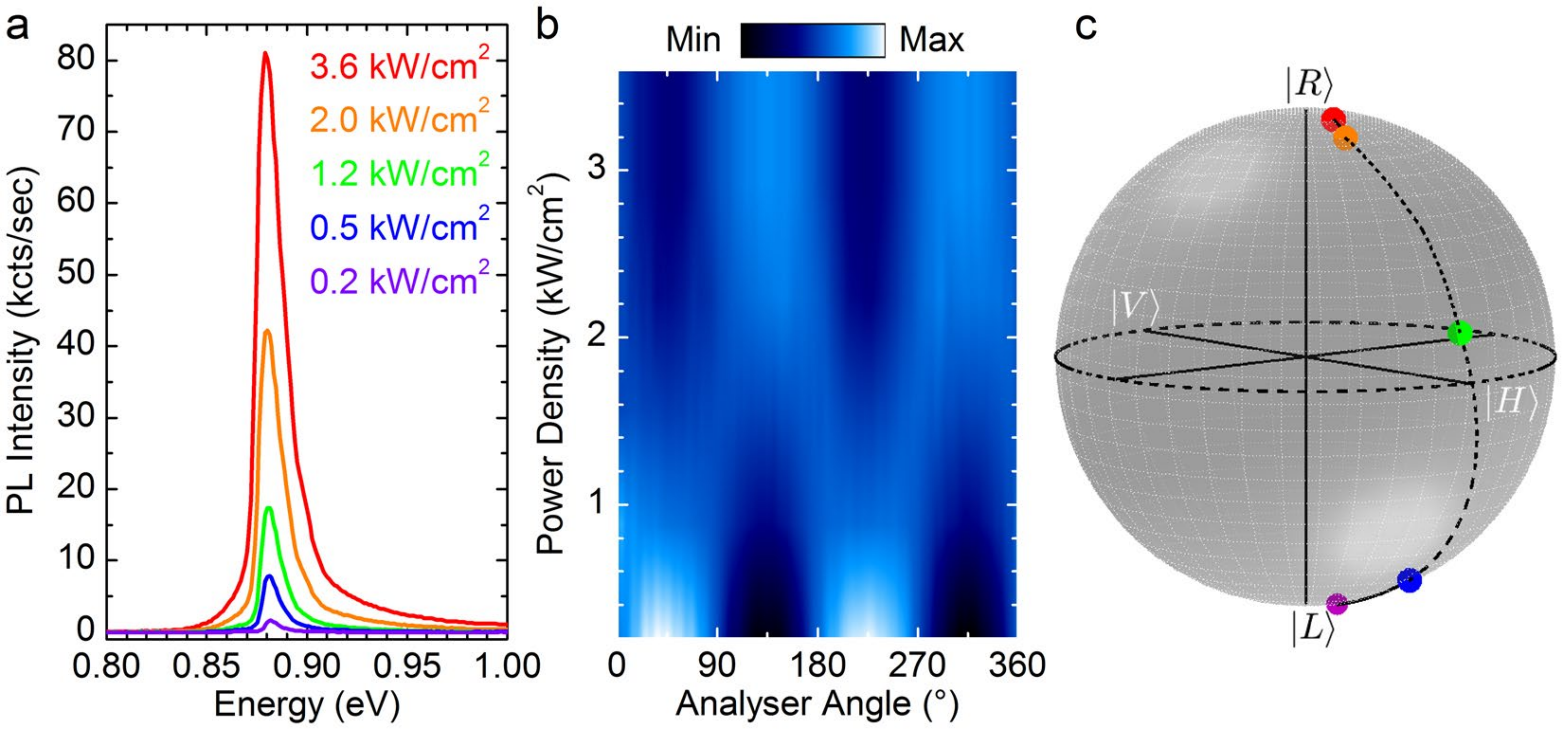
Power-dependent PL characteristics and Stokes analysis. (**a**) Direct gap photoluminescence (PL) spectra measured at 4 K and under various excitation power densities in a n-type bulk Ge wafer; (**b**) Color-coded map of the PL peak intensity as a function of the angle of the polarization analyser for different pump power densities. (**c**) Normalized Poincaré sphere of the polarized PL component as obtained by dividing the Stokes parameters derived from the modulation pattern of (**b**) by the total intensity of the light field^48^.

Figure 2a shows the direct gap PL under |*R*〉 excitation at 1.165 eV. The indirect gap PL occurs at a lower energy and will not be discussed in this work. All the measurements have been carried out at 4 K. The PL demonstrates a peak at ∼0.88 eV. The skewed lineshape stems from the joint density of states and the Maxwell-Boltzmann-like distribution of the carriers within their bands. The former determines the low energy threshold of the PL feature, while the latter yields an exponential-like tail on the high-energy side of the peak.

Figure 2a shows specifically the power-dependent PL characteristics. Besides the expected increase of the peak amplitude with the number of photoinjected carriers, the PL demonstrates a puzzling polarization pattern. Figure 2b reports a color-coded map of the normalized PL peak intensity as a function of the angle of a polarization analyser, namely a quarter wave plate followed by a linear polarizer. Figure 2b shows the data gathered for all the values of the incident power densities accessible in our experiment. Three well-defined regimes can be observed. At low pump power, the pattern reveals a sinusoidal behaviour with two maxima (minima) at 45° (135°) and 225° (315°). While the PL is partially polarized, this modulation unambiguously unveils a |*L*〉 polarization eigenstate, which is cross-polarized with respect to the |*R*〉 excitation^48,49^. In the high injection regime, however, the periodic feature is surprisingly out-of-phase with respect to the low power regime. Indeed, the light field turns out to be in the orthogonal |*R*〉 eigenstate, as demonstrated by the two maxima (minima) π-shifted at 135° (45°) and 315° (225°)^48,49^. Finally, at intermediate densities, the modulation frequency of the PL intensity oscillation demonstrates a twofold increase, heralding unambiguously a linearly polarized emission^48^. From the modulation pattern of Fig. 2b, we derived the Stokes vectors that have been normalized for the total intensity of the light field to provide an explicit and direct representation of the polarized component of the PL through the Poincaré sphere, as shown in Fig. 2c^48,49^. Stokes polarimetry clarifies the effectiveness of the incident power density, i.e. carrier population, in steering the polarized component of the PL along the sphere. Remarkably, a full helicity inversion of the polarization of the direct gap PL can be attained, eventually swapping the pure orthogonal |*R*〉 and |*L*〉 states.

Such subtle finding, which notably does not occur in intrinsic or heavily doped samples (see Supplementary Fig. 1), can be naturally understood in the framework of the electron spin dynamics taking place in a multivalley material, as described in the previous section. The larger the density of the photo-generated carriers, the larger the probability that electrons experience intravalley Coulomb-mediated collisions during the thermalization process.

### Kinetic model

In the following, we elaborate further on the key role of the free carrier density and Coulomb interactions in dictating the energy relaxation and eventually the PL polarization. To this purpose, we model the cooling processes experienced by hot electrons in the CB extending the three-state model proposed by Stanton and Bailey^50^. In particular, we explicitly include spin-dependent phenomena pertaining to a multi-valleyed material.

**Figure 3:**
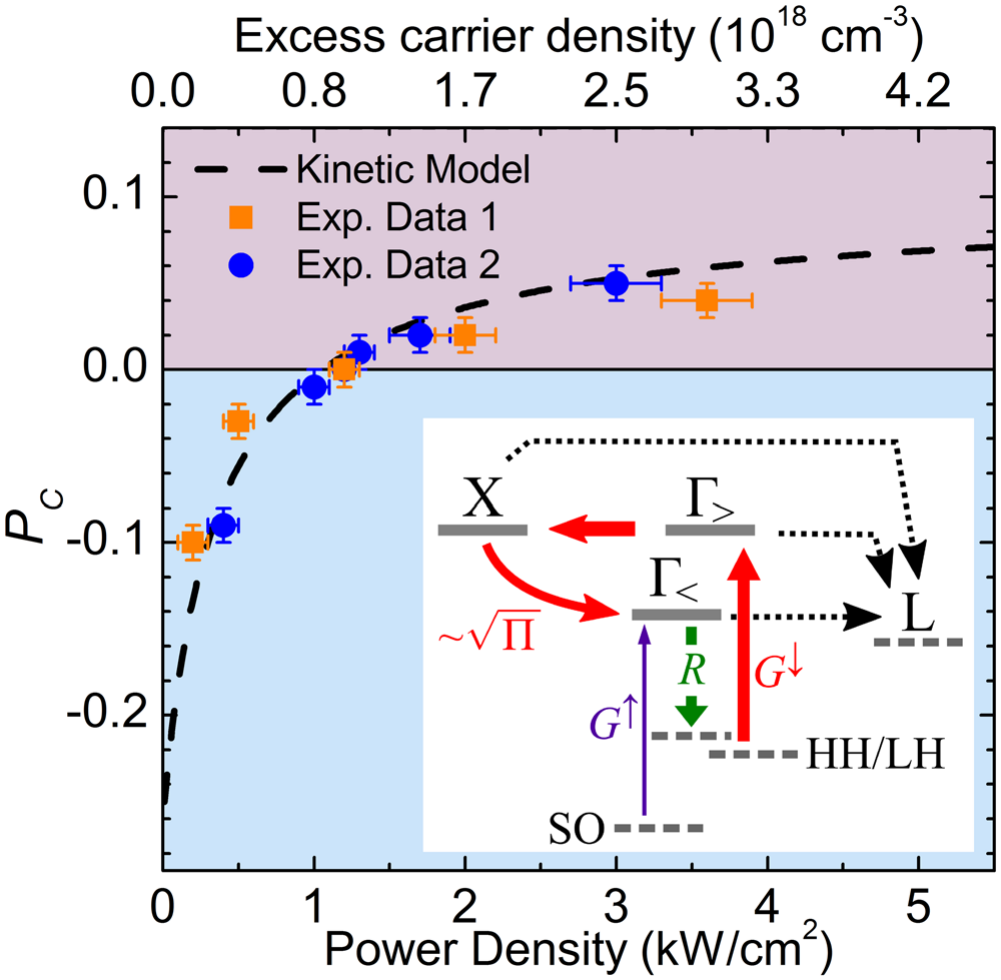
Modelling of the power-dependent PL polarization. Circular polarization degree of the direct gap PL measured as a function of the excitation power density in two n-doped Ge samples having the same doping content of 8.3 × 10^16^ cm^−3^ (red squares and blue circles). The estimated excess carrier density corresponding to the pump power is also shown (see Supplementary Note 2). The black dashed line is the result of the kinetic model obtained by considering the excitation and recombination dynamics of spin polarized carriers schematically shown in the inset. Under circularly polarized laser excitation, spin up electrons (*G*^↑^) are photoexcited close to the Γ valley edge (Γ_<_) from the split-off (SO) valence band state, while spin down electrons (*G*^↓^) are promoted from the heavy (HH) and light hole (LH) states to higher energies (Γ_>_). The indirect band gap nature of Ge favours scattering of electrons out of the zone centre towards the *X* valleys and the absolute *L* minima. However, increasing the excitation power density (П), it is possible to strengthen the *X-*Γ backwards scattering, thus enhancing the spin down electron population contributing to the direct gap polarized PL (*R*). The different arrows thicknesses suggest the different efficiencies of the processes.

The simplest system that we can consider is schematically shown in the inset of Fig. 3. It includes an energy level near the edge of the Γ valley, termed ${{\rm{\Gamma }}}_{ < }$, which is partly occupied by spin-up thermal electrons originated from the split-off VB. We assume that under steady state conditions, electrons can recombine radiatively through direct band gap transitions only via this state. A second higher energy level, ${{\rm{\Gamma }}}_{ > }$, hosts electrons optically coupled to the topmost VB states having a net spin down orientation. Finally, we consider a third level for the electrons being scattered to the *X* valleys. It should be noted that, due to the indirect nature of the Ge band structure, the absolute *L*-valley minima of the CB are included in the model as a sink in which carriers can recombine without contributing to the final polarization of the direct gap PL. The dynamics of the most relevant processes involving these states can be generalized as follows:

1 $$[\begin{array}{c}{\dot{n}}_{{{\rm{\Gamma }}}_{ > }^{\downarrow }}\\ {\dot{n}}_{{\rm{X}}}\\ {\dot{n}}_{{{\rm{\Gamma }}}_{ < }^{\downarrow }}\\ {\dot{n}}_{{{\rm{\Gamma }}}_{ < }^{\uparrow }}\end{array}]=[\begin{array}{cccc}-({\gamma }_{{\rm{\Gamma }}X}+{\gamma }_{{{\rm{\Gamma }}}_{ > }{\rm{L}}}) & 0 & 0 & 0\\ {\gamma }_{{\rm{\Gamma }}X} & -({\gamma }_{X{\rm{\Gamma }}}+{\gamma }_{{\rm{X}}{\rm{L}}}) & 0 & 0\\ 0 & {\gamma }_{X{\rm{\Gamma }}} & -(R+{\gamma }_{{{\rm{\Gamma }}}_{ < }{\rm{L}}}) & 0\\ 0 & 0 & 0 & -(R+{\gamma }_{{{\rm{\Gamma }}}_{ < }{\rm{L}}})\end{array}]\,[\begin{array}{c}{n}_{{{\rm{\Gamma }}}_{ > }^{\downarrow }}\\ {n}_{{\rm{X}}}\\ {n}_{{{\rm{\Gamma }}}_{ < }^{\downarrow }}\\ {\dot{n}}_{{{\rm{\Gamma }}}_{ < }^{\uparrow }}\end{array}]+[\begin{array}{c}{G}^{\downarrow }\\ 0\\ 0\\ {G}^{\uparrow }\end{array}]$$

where *n* refers to the non-equilibrium carrier density induced by the optical excitation, *γ*_*ij*_ is the net scattering rate from state *i* to *j*, *R* is the recombination rate, and *G*^↑,↓^ are the generation rates for spin up (↑) and spin down (↓) electrons, respectively. Further details about the model and its parameters are given in the Supplementary Note 1.

After light absorption, the ultrafast phonon-mediated scattering acts on the spin-down carriers at ${{\rm{\Gamma }}}_{ > }$, populating the *X* state. Coulomb-mediated interactions subsequently provide a very efficient energy loss channel^51,52^, eventually cooling the *X*-valley ensemble. The latter will then reach thermal equilibrium with the lattice by dwelling into the *L* valleys. Notably, an increase of the pump power strengthens the electron-plasmon coupling, as in a material having effectively a larger doping level. This process substantially accelerates the hot carrier relaxation^52,53^ and, in turns, contributes to the occurrence of scattering events that can guide some spin-down electrons towards the low-energy ${{\rm{\Gamma }}}_{ < }$ state^30^.

As an educated guess, we can therefore apply a linear approximation and expand the density-dependent *X*-to-Γ transition rate into a first order series, that is ${\gamma }_{X{\rm{\Gamma }}}\approx {\gamma }_{X{\rm{\Gamma }}}^{0}+{\gamma }_{X{\rm{\Gamma }}}^{1}\cdot {n}_{{\rm{X}}}$. As demonstrated in the Supplementary Note 1, where we derived an analytic expression for the populations of the ${{\rm{\Gamma }}}_{ < }$ level under the steady state approximation, the overall *X*-to-Γ backward scattering is governed by the pump power density, П, according to a square-root power law. In light of this finding, it can be expected that an increase of the pump fluency triggers an accumulation of a spin-down subset at the ${{\rm{\Gamma }}}_{ < }$ state, eventually modifying the circular polarization degree (*P*_*c*_) of the PL.

Figure 3 compares the *P*_*c*_(Π) curve predicted by the kinetic model with the circular polarization degree, ${P}_{C}=\frac{{I}^{|R\rangle }-{I}^{|L\rangle }}{{I}^{|R\rangle }+{I}^{|L\rangle }}$, experimentally determined at various excitation power densities by measuring the PL intensity I of the |*R*〉 and |*L*〉 components in two Ge:As samples having the same nominal doping content. For a better comparison, we also provide the photo-injected carrier density corresponding to the pump power level utilized in each measurement (for the details about such conversion see Supplementary Note 2). The striking agreement disclosed by Fig. 3 is an open evidence of the emergence of Coulomb coupling, particularly electron-plasmon interactions. It also demonstrates that the modelling, despite being simple, captures the physics of the spin-dependent carrier dynamics.

Finally, we note that, according to the theory, the pump power regime explored in this work results in ${\gamma }_{X{\rm{\Gamma }}}^{0}\gg {\gamma }_{X{\rm{\Gamma }}}^{1}\cdot {n}_{{\rm{X}}}$ in intrinsic or weakly doped samples, whereas the opposite holds for heavily doped Ge. Such constraints impede the observation of a power-induced helicity change at these extremal doping regimes, in nice agreement with the results shown in the Supplementary Fig. 1.

### Spin and energy distribution of Γ-electrons

Here we focus on the intermediate pump power regime in order to analyse in greater details the linearly polarized emission, and additionally test the physical picture emerged from the previous discussion.

**Figure 4:**
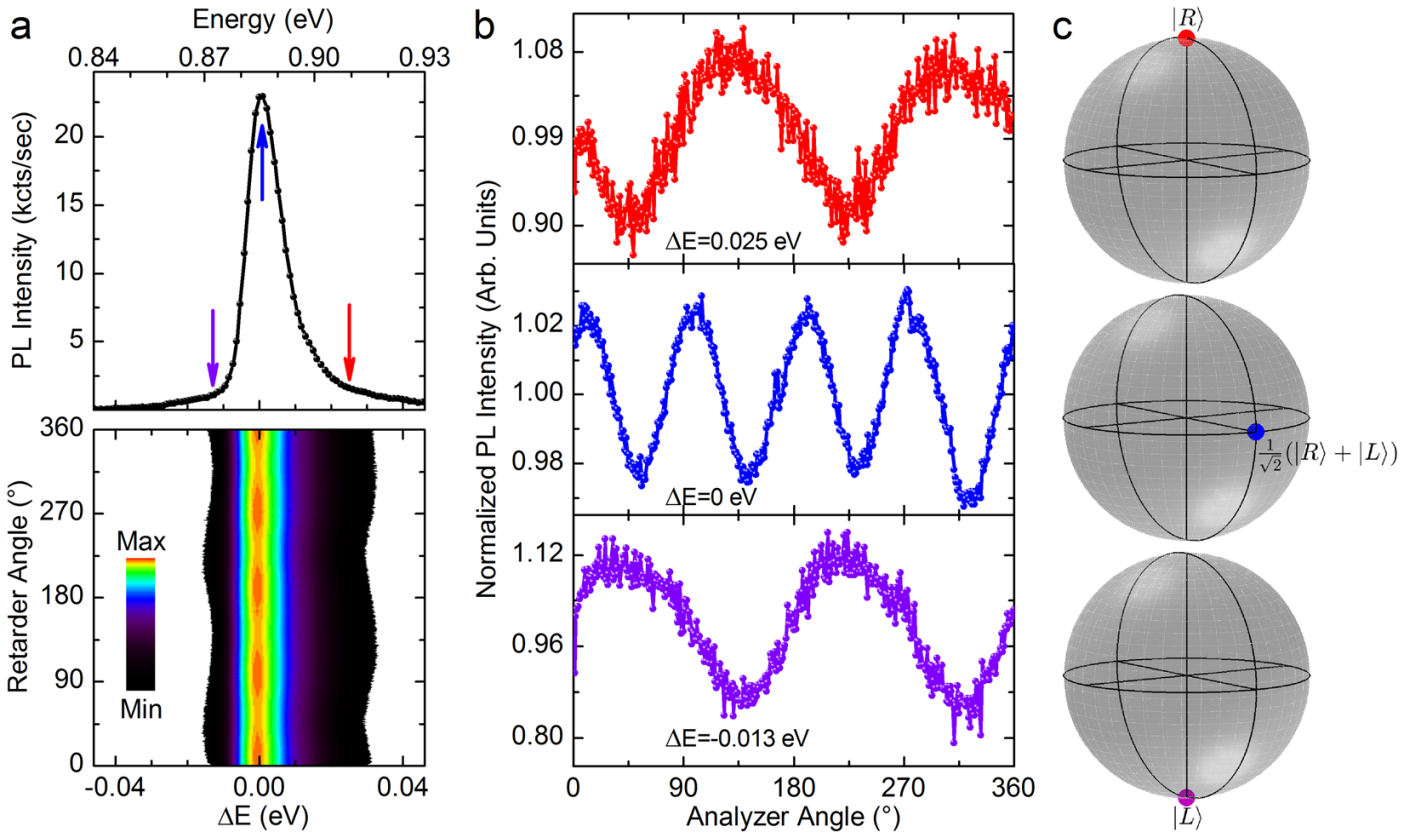
Polarization-resolved energy spectrum of Ge. (**a**) Low-temperature direct gap PL of Ge under 1.2 kW/cm^2^ excitation power density (upper panel). Color-coded map of the PL intensity describing the modulation of the direct-gap PL as a function of the analyser angle (lower panel). ΔE corresponds to the emission energy shifted with respect to the spectral position of the PL peak. (**b**) Modulation of the PL peak intensity as a function of the angle of the polarization analyser. The data are spectrally resolved for photons emitted at the PL peak (cyan curve), at a positive (red curve) and at negative (violet curve) energy detuning ΔE. (**c**) Qualitative representation on the Poincaré sphere of the polarized component of the PL corresponding to the ΔE values shown in b.

Figure 4a shows the detailed color-coded map of the modulation intensity of the PL peak for a value of the incident power density of about 1.2 kW/cm^2^. For convenience, the energy scale has been shifted with respect to the peak maximum, with the aim to better emphasize the PL contributions of carriers that differ for their kinetic energy. As previously discussed, a pump fluency of 1.2 kW/cm^2^ leads to the observation of linearly polarized emission at the peak energy, i.e. ΔE = 0 (see Fig. 2b and the middle panel of Fig. 4b).

A fine energy- and polarization-resolved analysis of the direct gap PL under this excitation condition unveils, however, a more elaborate and intriguing scenario. Indeed, a modest energy detuning of few tens of meV from the peak maximum remarkably demonstrates circularly polarized emission, see Fig. 4b,c. Chiefly, the high energy tail of the PL peak, i.e. ΔE > 0, yields a right-handed (|*R*〉) circular polarization eigenstate, whilst the low energy side (ΔE < 0) unambiguously discloses the pattern of a cross-polarized |*L*〉 eigenstate.

This evidence corroborates the modelling of the kinetics of the spin-polarized carriers. Above all, it clarifies that the linearly polarized photons emitted at the PL peak are due to a genuine superposition of the circular basis, namely ½(|*L*〉 + |*R*〉), stemming from the spin-resolved energy spectrum of the Γ-valley electrons. Besides further strengthening the physical picture of the optically reconfigurable polarization of the direct gap PL, the linear polarization is a direct manifestation that the fast spin-dependent relaxation pathways can yield a coherent superposition of two cross-polarized spin ensembles dwelling at the bottom of the Γ-valley.

## Discussion

We clarified the mechanisms that affect the energy relaxation of hot CB electrons in a technologically relevant material such as Ge. In particular, Coulomb interactions have been shown to reduce the ultrafast transfer rate towards the absolute minimum in the *L* valleys and enhance the backward scattering towards the zone centre. This permits to alter the relative weight of the two cross-polarized spin populations dwelling at Γ and, in turn, to define the one that dominates the recombination events across the direct gap. We thus demonstrated that the kinetics of spin-polarized electrons in a multivalley conduction band material, such as Ge, can be readily modified by simply varying the free-carrier density. This can possibly open viable routes to gather a better understanding of electron-plasmon coupling and hot charge-carrier interactions^54^, thereby providing relevant information for fields such as semiconductor plasmonics.

In view of the applications, the simple change of the intervalley scattering rate via the density of photogenerated carriers yields an effective modulation of the PL polarization without any external means, namely magnetic fields or optical retarders and can rival with recent proposals based on metamaterials^55–57^, polariton condensates^58,59^, atomically thin transition metal dichalcogenides^60,61^ and quantum dots^62,63^. In this context, the conceivable limit of the switching speed of the method originating from this work would be given by the ps-scale dynamics of those electrons that experience cooling into the *X* valleys^44^. This could potentially give rise to modulation frequencies unmatched by mechanically driven waveplates and faster than the operational frequencies of commercial photoelastic modulators. An additional benefit of the present approach is that it does not require high voltage electronics, which is otherwise mandatory to drive retarders such as Pockels cells. In the future, microcavities and photonic crystals can be also exploited to suitably tailor light-matter interaction and scrutinized as a means to increase the polarization degree of the emitted light.

The demonstration of tuneable polarization at the direct gap transition in Ge, although achieved at low temperatures and with low polarization values, can stimulate novel investigations aimed to implement spin-optoelectronic functionalities directly in a CMOS-compatible platform^64^. Above all, the proposed concept of optically adjustable spin populations can be extended to other materials and epitaxial heterostructures. It is worth noting that GeSn binary alloys have just been explored as direct gap materials that can be epitaxially grown on Si substrates^35^. It can be thus expected that SiGeSn alloys would soon lead to a wide and yet untapped range of multi-valleyed electronic structures^65^, which can benefit from the guidance of our investigation to implement efficient and ultracompact emitters boasting a high polarization degree^66^.

## Methods

### Experimental Setup

PL measurements were carried out in a backscattering geometry using a closed-cycle cryostat. The working temperature was 4 K and the incident power density was ranging between ∼10^−1^ and 10 kW/cm^2^. The samples were excited by a continuous wave Nd:YVO_4_ laser operating at 1.165 eV. The incident light was right-handed circularly polarized |*R*〉, and the laser spot on the sample surface has a Gaussian shape with a full width at half maximum of (60 ± 3) µm. The polarization state of emitted light was probed by means of a quarter-wave plate (λ/4 ± 4° in the 700–2500 nm range) followed by a linear polarizer having a contrast larger then 10^6^:1 in the 900–1550 nm spectral window. The amplitude of the PL peak as a function of the analyser angle was measured by using a spectrometer equipped with an InGaAs array multiple-channel detector with a cut-off energy at about 0.755 eV. The energy accuracy of the whole system is of about 4 meV. Stokes parameters were provided by the analysis of peak amplitude modulation, allowing the characterization of the state of light polarization^48,49^.

### Data availability

The datasets generated during and/or analysed during the current study are available from the corresponding author on reasonable request.

## Electronic supplementary material

Supplementary Information
