# Supplementary material for: Optically reconfigurable polarized emission in Germanium
Source: Sci Rep. 2018 Jul 24;8:11119. doi: 10.1038/s41598-018-29409-3 (PMC6058013; doi:10.1038/s41598-018-29409-3)
Supplement: Supplementary file 1 — Supplementary Information [file 41598_2018_29409_MOESM1_ESM.pdf]

Optically reconfigurable polarized emission in Germanium

Sebastiano De Cesari, Roberto Bergamaschini, Elisa Vitiello, Anna Giorgioni, and Fabio Pezzoli

LNESS and Dipartimento di Scienza dei Materiali, Università di Milano-Bicocca, via Cozzi 55, 20125 Milano, Italy

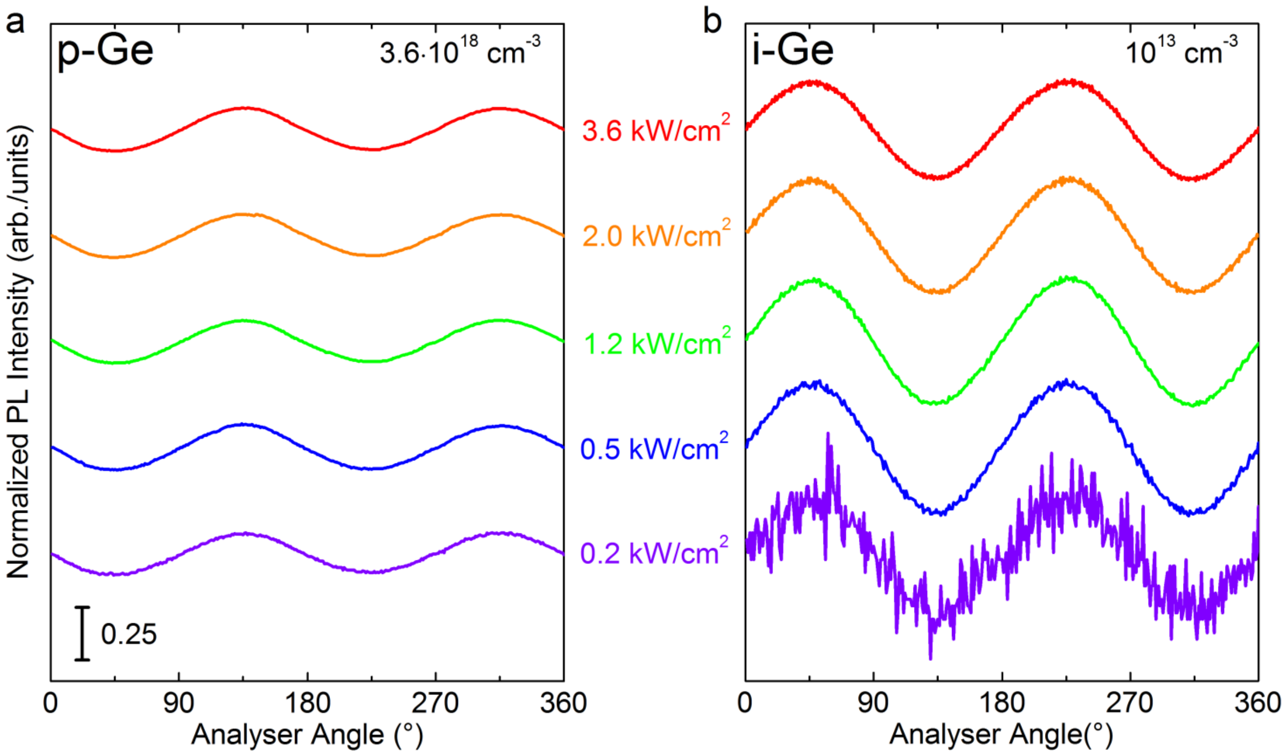

Supplementary Figure 1| PL polarization of Ge samples with high and low doping content. Low temperature PL peak intensity modulations as a function of the angle of the polarization analyser. Measurements are relative to a p-type Ge:Ga wafer, with an acceptor concentration of 3.6×10^{18} cm^{-3} (panel a) and an intrinsic Ge sample, with a resistivity of 47 Ω cm, named i-Ge (panel b). The former demonstrates a $|R\rangle$ while the latter a $|L\rangle$ polarization eigenstates. Remarkably, none of the samples demonstrates a change in helicity upon varying the pump power. The circular polarization degree (P_C) lies between -0.23 and -0.3 for the i-Ge, while for p-Ge $+0.10 \leq P_C \leq +0.11$.

Supplementary Note 1. Kinetic model. A three-state system is taken into account. It is based on a state at the bottom of the Γ valley, $\Gamma_<$, from which carriers recombine radiatively. A second higher energy level, $\Gamma_>$, hosts electrons photo-excited from the top of the valence band. A third additional state is assumed to exist in the X valleys. Owning to the indirect band gap of Ge, the majority of electrons accumulates at the L valleys and thereby do not contribute to the zone-centre radiative recombination.

All the relevant processes between these states result in the following rate equations:

$$\begin{cases} \dot{n}_{\Gamma_>}(\downarrow) = G^{\downarrow} - \left(\gamma_{\Gamma X} + \gamma_{\Gamma_> L}\right) \cdot n_{\Gamma_>}(\downarrow) \\ \dot{n}_{X} = \gamma_{\Gamma X} \cdot n_{\Gamma_>}(\downarrow) - (\gamma_{X\Gamma} + \gamma_{XL}) \cdot n_{X} \\ \dot{n}_{\Gamma_<}(\downarrow) = \gamma_{X\Gamma} \cdot n_{X} - \left(R + \gamma_{\Gamma_< L}\right) \cdot n_{\Gamma_<}(\downarrow) \\ \\ \dot{n}_{\Gamma_<}(\uparrow) = G^{\uparrow} - \left(R + \gamma_{\Gamma_< L}\right) \cdot n_{\Gamma_<}(\uparrow) \end{cases} \quad (1)$$

where n refers to the excess electron density induced by the optical excitation with respect to the intrinsic level and γ_{ij} is the net scattering rate from state i to j. Scattering rates are set as in Supplementary Table 1. As the X→Γ transition is believed to be significantly affected by the Coulomb scattering between the photo-generated electrons and the background carriers, by a first order approximation, the transition rate $\gamma_{X\Gamma}$ is modelled as linearly increasing with the population of electrons in the X state, i.e. $\gamma_{X\Gamma} = \gamma_{X\Gamma}^{0} + \gamma_{X\Gamma}^{1} \cdot n_{X}$. The two unknown parameters, namely $\gamma_{X\Gamma}^{0}$ and $\gamma_{X\Gamma}^{1}$, are derived from the experimental data (see below).

The first three equations in (1) trace the relaxation path of the spin-down high-energy electrons promoted in the $\Gamma_>$ state by photo-generation with rate $G^{\downarrow}$. The latter approximately corresponds to the 95% of the overall generation rate $G = \alpha(h\nu)^{-1}\Pi$ (with $\alpha = 16321\ cm^{-1}$ being the absorption coefficient of Ge at $h\nu$= 1.16 meV as the laser excitation energy). The cooling to the $\Gamma_<$ state occurs via the X state, with the yield reduced by the scattering towards the L valleys. The last equation in (1) sets the population of the $\Gamma_<$ states by spin-up polarized electrons generated from the SO band, with rate $G^{\uparrow}$ ≈0.05×G. Electrons in the $\Gamma_<$ band bottom can recombine radiatively with rate R or be

Literature Ref. [1]	$\gamma_{\Gamma_{>}\mathrm{X}}$	$28\ ps^{-1}$
	$\gamma_{\Gamma_{>}\mathrm{L}}$	$9.3\ ps^{-1}$
	γ_{XL}	$9.0\ ps^{-1}$
	$\gamma_{\Gamma_{<}L}$	$5.3\ ps^{-1}$
Fit from exp.	$\gamma_{\mathrm{X}\Gamma} = \gamma_{\mathrm{X}\Gamma}^{0} + \gamma_{\mathrm{X}\Gamma}^{1} \cdot n_{\mathrm{X}}$	
	$\gamma_{\mathrm{X}\Gamma}^{0}$	$0.30\ ps^{-1}$
	$\gamma_{\mathrm{X}\Gamma}^{1}$	$(32\ \pm 3)\ (h\nu)/\alpha\ ps^{-1}cm^{3}$

Supplementary Table 1| Intervalley scattering rates. Scattering rates, γ_{ij}, between the valleys Γ, *X* and *L* in Ge as derived from Ref. 1 for an initial excess energy of the photogenerated electrons corresponding to about 200-300 meV. The back-scattering rates, $\gamma_{\mathrm{X}\Gamma}^{0}$ and $\gamma_{\mathrm{X}\Gamma}^{1}$, between the valleys Γ and *X* have been used for the integration of the rate equations shown in Eq (1).

scattered toward the *L* valleys. The relative $\Gamma_{<}/\Gamma_{>}$ population upon light absorption has been derived from a recent literature work dealing with Ge [2].

Due to the final spin polarization of the electron population at the $\Gamma_{<}$ level, determined by the relative efficiency of the relaxation channels, the emitted light has a net circular polarization degree P_c. As shown in Ref. [2], photo-generated electrons are partially polarized: ~30% for the spin-down electrons and ~90% for the spin-up population. We can therefore conclude that

$$P_c \sim \frac{0.3\ n_{\Gamma_{<}}(\downarrow) - 0.9\ n_{\Gamma_{<}}(\uparrow)}{2\left(n_{\Gamma_{<}}(\downarrow) + n_{\Gamma_{<}}(\uparrow)\right)} \qquad (2)$$

where the 1/2 factor accounts for the spin depolarization resulting from the radiative recombination with the unpolarized holes at the top of the VB.

An analytic expression for the populations of the $\Gamma_{<}$ level can be derived by solving the rate equations in (1) under the stationary conditions

$$P_c(I) = \frac{0.3\left[AG^{\downarrow}\Pi + B\gamma_{XL}\left(1-\sqrt{1+\frac{A}{\tilde{\gamma}^1_{\mathrm{X\Gamma}}B^2}\cdot G^{\downarrow}\Pi}\right)\right] - 0.9G^{\uparrow}\Pi}{2\left[AG^{\downarrow}\Pi + B\gamma_{XL}\left(1-\sqrt{1+\frac{A}{\tilde{\gamma}^1_{\mathrm{X\Gamma}}B^2}\cdot G^{\downarrow}\Pi}\right) + G^{\uparrow}\Pi\right]} \quad (3)$$

with $\tilde{\gamma}^1_{\mathrm{X\Gamma}} = \gamma^1_{\mathrm{X\Gamma}}\alpha(h\nu)^{-1}$, $A = \frac{\gamma_{\Gamma>X}}{\gamma_{\Gamma>L}+\gamma_{\Gamma>X}}$ and $B = \frac{\gamma^0_{\mathrm{X\Gamma}}+\gamma_{XL}}{2\tilde{\gamma}^1_{\mathrm{X\Gamma}}}$. In particular, it can be shown that

$$\gamma_{\mathrm{X\Gamma}} = \frac{\gamma^0_{\mathrm{X\Gamma}}-\gamma_{XL}}{2} + \frac{\gamma^0_{\mathrm{X\Gamma}}+\gamma_{XL}}{2}\sqrt{1+\frac{2A}{B}\cdot G^{\downarrow}\Pi} \quad (4)$$

and therefore the excitation of spin-down electrons is expected to dominate when the pump power is above a certain threshold, as observed in the experiments. Notice that the degree of polarization of the emitted light $P_c(\Pi)$ does not depend on the actual rate R of the recombination process from which it originates because it is assumed to be the same for both cross-polarized spin $\Gamma_<$ ensembles. For the same reason, $P_c(\Pi)$ is also unaffected by the $\gamma_{\Gamma_< \mathrm{L}}$ transition rate. Finally, according to eq. (3), in order to obtain a variation of the polarization P_c with respect to the excitation power density, Π, a non-zero transition rate γ_{XL} is required.

To quantify the different kinetics of the spin-polarized carriers, $\gamma^0_{\mathrm{X\Gamma}}$ is estimated by considering the light polarization resulting in the limit of zero-power excitation (Π→0), reasonably corresponding to the case of intrinsic Ge. An average value of -0.26 for the circular polarization degree is measured (see Supplementary Figure 1), thus providing a value of $\gamma^0_{\mathrm{X\Gamma}} = 0.30\ ps^{-1}$. Then $\gamma^1_{\mathrm{X\Gamma}}$ is obtained by fitting the theoretical curve to the experimental data, resulting in $\gamma^1_{\mathrm{X\Gamma}} \approx (32 \pm 3)\ (h\nu)/\alpha\ ps^{-1}cm^3$. The very good agreement between the theoretical curve and the experimental data, even if relying on overly simplified assumptions, corroborates the idea that Coulomb interactions play a crucial role in determining the carrier kinetics. Nonetheless, a proper quantitative determination of the X→Γ transition rate is out of the scope of the present work, in view of the phenomenological approach here exploited. Still, it is notable that a reasonable correspondence of the fitted curve to the experimental data remains valid even by varying the fitting parameter by a ±30%, as illustrated by the shadowed

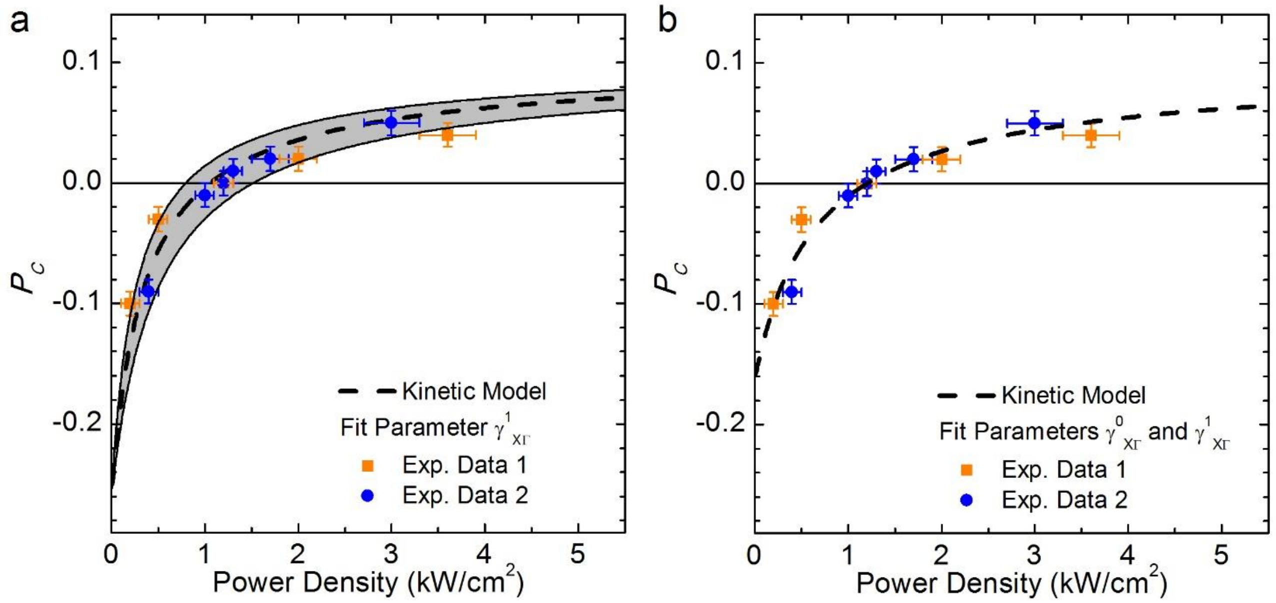

Supplementary Figure 2| Model's predictivity. a Circular polarization degree, P_c, as a function of the excitation power density Π. The model is shown as a dashed line, while the orange and blue symbols correspond to the two experimental datasets obtained on Ge:As wafers whose doping concentration is 8.3×10^{16} cm^{-3} (dots). The model utilizes $\gamma^1_{X\Gamma}$ as a fitting parameter, while $\gamma^0_{X\Gamma}$ has been chosen to reproduce P_c=-0.26 in the limit of zero-power excitation. The gray region shows a confidence region for the fit corresponding to a ±30% variation of $\gamma^1_{X\Gamma}$. **b** P_c versus Π obtained by utilizing both $\gamma^0_{X\Gamma}$ and $\gamma^1_{X\Gamma}$ as fit variables.

area in the Supplementary Figure 2a. This supports the conclusion that the qualitative behaviour expected from the simple model is not critically dependent on the accuracy of $\gamma^1_{X\Gamma}$. As a further test, we released the constraint on $\gamma^0_{X\Gamma}$. The results are shown in the Supplementary Figure 2b. In this case, the best fit is given by $\gamma^0_{X\Gamma} = 0.62\, ps^{-1}$ and $\gamma^1_{X\Gamma} = 24\, (h\nu)/\alpha\, ps^{-1}cm^3$. However, the limit of zero-power excitation would imply a value of P_c=-0.16, which is 40% smaller than the most likely experimental value to be expected, i.e. the one derived from the measurement of the intrinsic Ge sample. Nonetheless, the possibility to satisfactorily reproduce the experimental behaviour even by this second method further substantiate the robustness of our phenomenological approach and likewise the accuracy of the physical picture which leads to the $\sqrt{\Pi}$ dependence.

Supplementary Note 2. Determination of the photoinjected carrier density.

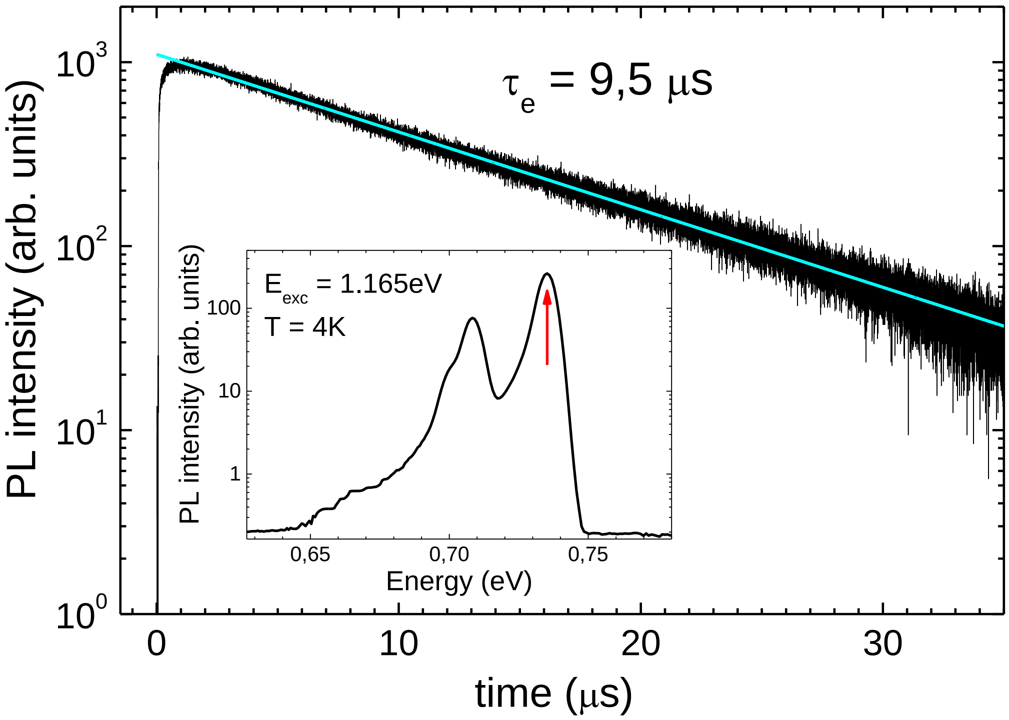

Supplementary Figure 3| Time decay of the indirect gap photoluminescence. Decay curve obtained at 4K for the indirect gap phonon-mediated photoluminescence (PL). Data refers to the highest energy peak at about 0.735 eV as observed in the continuous-wave low temperature PL spectrum shown in the inset. The blue solid line is a fit of the decay curve. A decay time of 9.5 μs has been derived by utilizing a single exponential decay.

In order to provide a simple estimate for the total concentration of the excess carriers, *n*, corresponding to the various excitation power densities, Π, exploited in this work, we utilize the following simplified one-dimensional continuity equation:

$$\frac{\partial n}{\partial t} = G - \frac{n}{\tau_e} \quad (5)$$

where the overall generation rate, *G*, has been determined according to the infrared excitation condition as described in the Supplementary Note 1. As shown in Supplementary Figure 3, the effective carrier lifetime, τ_e, in the Ge:As samples having an impurity concentration of 8.3×10^{16}cm^{-3}

has been determined to be 9.5 µs. In this case, time-correlated single photon counting experiments were carried out using a Nd:YAG Q-switched laser at 10 kHz frequency as discussed in the supplementary Ref. 3. Finally, under stationary condition, Equation (5) can lead to the following formula linking the photogenerated carrier density, expressed in cm^{-3}, to the pump power density, expressed in kW/cm^2:

$$n\,[\mathrm{cm}^{-3}] = G \times \tau_e = 8.35 \times 10^{17}\,\Pi[\mathrm{kW/cm}^2] \qquad (6)$$

Supplementary References

1. Bailey D W and Stanton C J. Calculations of femtosecond differential optical transmission in germanium. J. Appl. Phys. 77, 2107 (1995).
2. Vitiello E, Virgilio M, Giorgioni A, Frigerio J, Gatti E, De Cesari S, Bonera E, Grilli E, Isella G and Pezzoli F. Spin-dependent direct gap emission in tensile-strained Ge films on Si substrates. Phys. Rev. B 92, 201203 (2015).
3. Giorgioni A, Paleari S, Cecchi S, Vitiello E, Grilli E, Isella G, Jantsch W, Fanciulli M and Pezzoli F. Strong confinement-induced engineering of the g factor and lifetime of conduction electron spins in Ge quantum wells. Nat. Commun. 7, 13886 (2016).
